# Endobiliary metastasis of colorectal cancer: A rare presentation and diagnostic dilemma: A case report

**DOI:** 10.1016/j.ijscr.2025.111192

**Published:** 2025-03-21

**Authors:** Guelbi Mohamed, Hajri Mohamed, Tangour Monia, Ghariani Rayfa, Bayar Rached, Bacha Dhouha

**Affiliations:** a Department of Surgery, Mongi Slim Hospital, Marsa, Tunisia; b Department of Pathology, Mongi Slim Hospital, Marsa, Tunisia; c Department of Radiology, Mongi Slim Hospital, Marsa, Tunisia

**Keywords:** Surgery, Liver, Cancer, Colorectal, Biliary, Case report

## Abstract

**Introduction:**

Colorectal cancer is a common malignancy with liver metastases frequently encountered. However, endobiliary metastases are extremely rare and can pose significant diagnostic challenges as they can mimic primary biliary tumors.

**Presentation of case:**

We report a 66-year-old female who underwent an extended right hemicolectomy for a well-differentiated adenocarcinoma of the transverse colon pT4aN1bM0. Two years after surgery, follow-up imaging revealed intrahepatic biliary dilation and subtle intraductal lesions, along with suspicious splenic nodules. Hepatic MRI confirmed the presence of endoluminal biliary nodules with upstream dilation. Percutaneous biopsy and immunohistochemical analysis showed tumor cells positive for CDX2 and negative for CD20, supported the diagnosis of intrabiliary colonic metastasis.

**Discussion:**

The clinical and radiological features of endobiliary metastasis can mimic those of primary biliary neoplasms, making accurate diagnosis challenging. While imaging modalities such as multiphasic CT and MRI are invaluable for lesion characterization, definitive diagnosis relies on tissue sampling and immunohistochemical profiling. Recognizing the possibility of endobiliary metastasis in patients with a history of colorectal cancer is crucial for appropriate management.

**Conclusion:**

Increased awareness of endobiliary metastasis as a manifestation of colorectal cancer metastasis is essential for differentiating it from primary biliary tumors, optimizing therapeutic strategies, and ultimately improving patient outcomes.

## Highlights

• Endobiliary metastasis of colorectal cancer is an extremely uncommon phenomenon, often leading to diagnostic challenges.
• The radiological features can mimic primary biliary neoplasms like cholangiocarcinoma.
• Confirmatory diagnosis relies on immunohistochemical markers which help differentiate it from primary biliary tumors.
• A multidisciplinary approach is essential for accurate diagnosis and effective management.

## 1 Introduction

Colorectal cancer (CRC) is one of the most common malignancies ranking third worldwide [1], with liver being the primary metastatic site [2]. However, endobiliary metastasis of CRC is a rare and often underrecognized phenomenon with an incidence as low as 3,6 % [3] making its diagnosis and management a challenge. Through this case we aim to provide a clinical presentation, diagnostic strategies, and management options for endobiliary metastasis of colorectal cancer.

## 2 Presentation of case

We report the case of a 66-year-old female patient with no prior medical history, she was operated for a transverse colon tumor she had an extended right hemicolectomy. Histopathological examination of the specimen revealed a well differentiated adenocarcinoma of the colon classified pT4aN1b.No distant metastases were detected by the pre-operative CT scan of chest and abdomen. The patient received an adjuvant chemotherapy (XELOX 6 cycles) and was regularly monitored in our outpatient department.

**Fig. 1:**
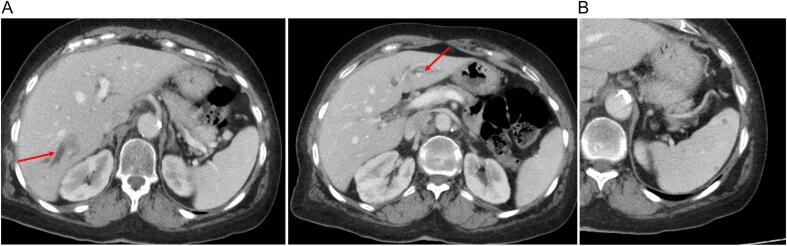
A: Axial abdominal CT scan with portal time injection showing a dilation of biliary tracts of liver segments II and VI. B: Axial abdominal CT scan with portal time injection showing multiples low dense lesion of the spleen.

**Fig. 2:**
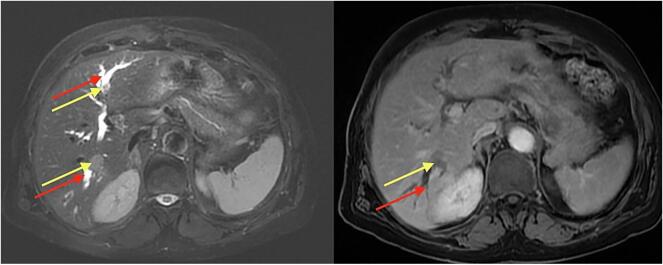
MRI of the liver with and without gadolinium injection showing a dilation of biliary tracts (red arrow) upstream two endoluminal biliary nodules in segments VII and II (yellow arrow). (For interpretation of the references to colour in this figure legend, the reader is referred to the web version of this article.)

During follow up, the patient was asymptomatic with a soft abdomen on clinical examination, laboratory test especially liver enzymes and tumor markers ACE and CA19-9 were in normal range. However, the CT scan after two years of the surgery revealed intrahepatic biliary duct dilation particularly in segments VI and II of the liver, with subtle low dense lesions in these bile ducts (Fig. 1A). The CT scan revealed also multiple millimetric nodules of the spleen (Fig. 1B). Hepatic MRI showed two endoluminal biliary nodules in segments VII and I, measuring 30 mm and 10 mm respectively with upstream biliary duct dilation (Fig. 2), suggestive of secondary lesions with multiple metastatic splenic nodules.

The patient underwent a percutaneous biopsy of the hepatic lesions, histopathological and immuhistochemical findings confirmed the presence of adenocarcinoma cells of primary colonic origin.

**Fig. 3:**
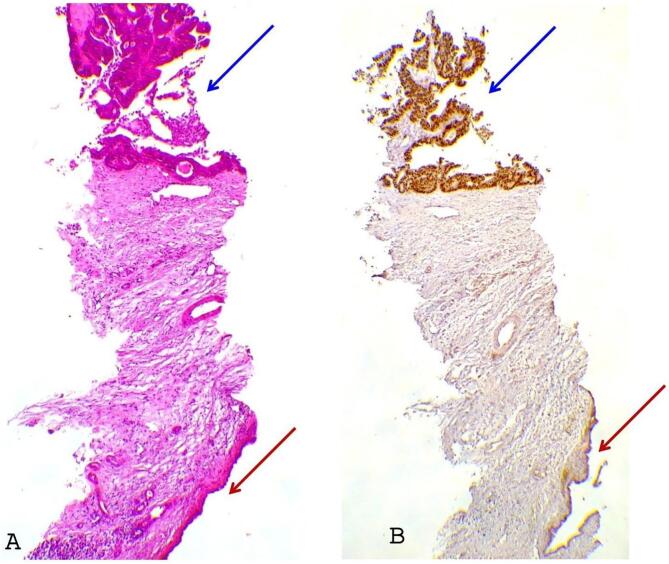
A: Microscopic examination. Tubulo-papillary carcinomatous expansion (blue arrow) budding in intra-lumen bordered by a biliary epithelium (red arrows) (hematoxylin-eosin: HE×100). B: Immunohistochemistry. Tubulo-papillary carcinomatous expansion (blue arrow) shows nuclear staining with CDX2 supporting the diagnosis of intestinal/colonic epithelium, while the biliary one (red arrows) is negative. (For interpretation of the references to colour in this figure legend, the reader is referred to the web version of this article.)

The standard hematoxylin eosin-stained slides showed tubulo-papillary carcinomatous expansion budding in intra-lumen bordered by a biliary epithelium (Fig. 3A), the neoplastic cells were all positive for CDX2 and negative for CD20 supporting the diagnosis of intrabiliary colonic metastasis (Fig. 3B). After multidisciplinary discussion it was decided to put the patient under chemotherapy (FOLFIRINOX).

## 3 Discussion

Endobiliary metastasis of colorectal cancer remains an exceedingly rare clinical entity, presenting significant diagnostic challenges. Its infrequent occurrence often means that clinicians may not initially consider it in the differential diagnosis of biliary obstruction and may be easily misdiagnosed with a primitive biliary or hepatic neoplasm on morphologic and clinical grounds. The history of colorectal cancer must be taken into consideration in patients presenting with dilated biliary ducts [4].

The pathophysiology behind endobiliary metastasis remains unclear, it has been hypothesized that tumor cells implantation in bile duct epithelium could be through a peribiliary capillary plexus communicating with portal veins and hepatic arteries [5].

As in our case, the clinical examination and routine laboratory tests are typically nonspecific. Patients may present with signs of biliary obstruction, such as jaundice or abdominal pain, and laboratory investigations might only reveal elevated liver enzymes with cholestasis, especially in cases involving proximal bile ducts, which represent 50 % of occurrences [6]. These findings are common to a broad spectrum of biliary disorders, thereby underscoring the need for a comprehensive diagnostic approach.

Radiological imaging is paramount in evaluating patients with suspected endobiliary metastasis. A multiphasic CT scan with liver protocol often reveals upstream biliary dilation or even subtle, hypodense intraductal lesions, these findings can mimic other biliary pathologies, such as primary cholangiocarcinoma [7]. Magnetic resonance imaging (MRI), with its superior soft-tissue contrast and the additional information provided by Diffusion-Weighted Imaging and Magnetic resonance cholangiopancreatography (MRCP), can help in the differential diagnosis of these challenging lesions [8]. Despite these advantages, imaging findings can often be ambiguous, as there is significant overlap in the radiological appearances of benign, primary malignant, and metastatic biliary lesions. This limitation further reinforces the need for additional diagnostic confirmation.

Therapeutic approaches to endobiliary metastasis of colorectal cancer are still evolving. The decision-making process should be tailored to each patient based on tumor burden, liver function, and overall performance status. Hepatectomy with a 1 mm margin is considered an adequate oncologic resection for colorectal liver metastasis [9] [10], while anatomical resection is necessary for a primary cholangiocarcinoma or a endobiliary metastasis of colorectal cancer [11]. However, for patients with non-resectable disease, systemic chemotherapy, remains the mainstay of treatment, aiming for tumor control and prolonged survival.

For cases where biliary obstruction leads to significant symptoms, palliative interventions such as endoscopic or percutaneous biliary drainage may be necessary to relieve obstruction and improve quality of life. The role of targeted therapies and immunotherapy in managing endobiliary metastases is an area of ongoing research, with emerging data suggesting that molecular profiling of tumors may guide personalized treatment strategies.

The unusual presentation in our case pushed us to complete the investigation by immunohistochemical analysis which plays a very important role in these cases. Tissue sampling obtained via percutaneous biopsy allows for the application of specific immunohistochemical markers that can conclusively differentiate colorectal metastases from primary biliary tumors. Markers such as CDX2 and CK20 are typically expressed in colorectal cancer cells and, when identified in biliary lesions, strongly indicate a colorectal origin [12]. The rarity of endobiliary metastasis from colorectal cancer, coupled with the nonspecific nature of clinical and laboratory findings, necessitates a high degree of clinical suspicion and a meticulous diagnostic approach. Patients' history should bring attention to this differential diagnosis, while radiological imaging plays a role in narrowing the diagnosis, it is ultimately the immunohistochemical analysis that provides the definitive diagnosis.

## 4 Conclusion

By improving awareness of this rare metastatic entity, clinicians can better differentiate it from other biliary pathologies and optimize patients' outcomes. Further research is essential to understand the pathophysiology behind it to optimize management strategies for this rare metastatic presentation.

## Author contribution

Mohamed Guelbi: conceptualization, data curation, redaction, project manager.

Mohamed Hajri: conceptualization, redaction.

Monia Tangour: resources, visualization.

Rayfa Ghariani: resources, visualization.

Rached Bayar: supervision, validation, visualization.

Dhouha Bacha: supervision, validation, visualization.

## Consent for publication

Written informed consent was obtained from legal authorized representatives before the study. On request, a copy of the written consent is available for review by the Editor-in-Chief of this journal.

## Ethical approval

Ethical approval is exempt/waived at our institution.

## Guarantor

Guelbi Mohamed.

## Research registration number

Not applicable.

## Funding

This work is not funded.

## Conflict of interest statement

The authors declare no competing interest.

## Data Availability

This published article includes all the required data.
